# *QuickStats:* Management of Patient Health Information Functions* Among Office-Based Physicians With and Without a Certified Electronic Health Record (EHR) System^†^ — National Electronic Health Records Survey, United States, 2018

**DOI:** 10.15585/mmwr.mm6938a8

**Published:** 2020-09-25

**Figure:**
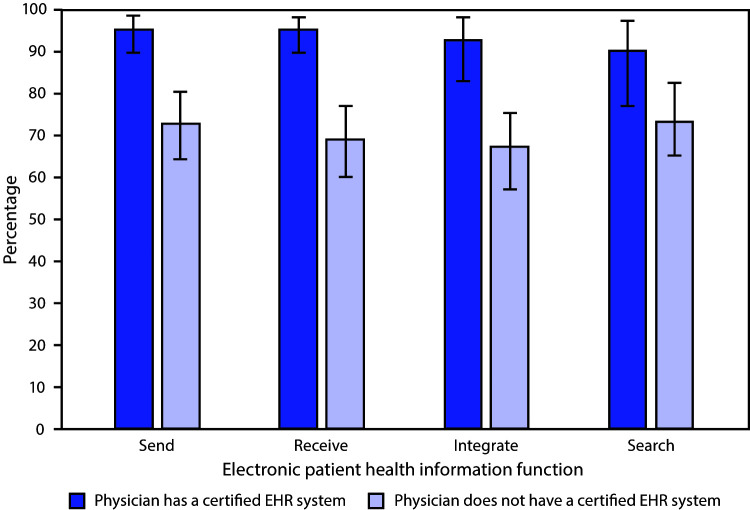
In 2018, 78.7% of office-based physicians had a certified electronic health record (EHR) system. A higher percentage of office-based physicians with a certified EHR system compared with those without a system electronically sent (95.5% versus 72.8%), received (95.3% versus 69.0%), integrated (92.8% versus 67.4%), or searched for (90.5% versus 73.3%) patient health information.

**Source:** National Electronic Health Records Survey, 2018. https://www.cdc.gov/nchs/nehrs/about.htm.

